# Connexins in Astrocyte Migration

**DOI:** 10.3389/fphar.2019.01546

**Published:** 2020-01-15

**Authors:** Raúl Lagos-Cabré, Francesca Burgos-Bravo, Ana María Avalos, Lisette Leyton

**Affiliations:** ^1^Cellular Communication Laboratory, Programa de Biología Celular y Molecular, Instituto de Ciencias Biomédicas (ICBM), Facultad de Medicina, Universidad de Chile, Santiago, Chile; ^2^Advanced Center for Chronic Diseases (ACCDiS), Center for Studies on Exercise, Metabolism and Cancer (CEMC), Facultad de Medicina, Instituto de Ciencias Biomédicas (ICBM), Universidad de Chile, Santiago, Chile; ^3^Facultad de Ciencias de la Salud, Instituto de Ciencias Biomédicas, Universidad Autónoma de Chile, Santiago, Chile

**Keywords:** connexin 43, gap junctions, hemichannels, inflammation, scar-forming astrocytes, reactive astrocytes

## Abstract

Astrocytes have long been considered the supportive cells of the central nervous system, but during the last decades, they have gained much more attention because of their active participation in the modulation of neuronal function. For example, after brain damage, astrocytes become reactive and undergo characteristic morphological and molecular changes, such as hypertrophy and increase in the expression of glial fibrillary acidic protein (GFAP), in a process known as astrogliosis. After severe damage, astrocytes migrate to the lesion site and proliferate, which leads to the formation of a glial scar. At this scar-forming stage, astrocytes secrete many factors, such as extracellular matrix proteins, cytokines, growth factors and chondroitin sulfate proteoglycans, stop migrating, and the process is irreversible. Although reactive gliosis is a normal physiological response that can protect brain cells from further damage, it also has detrimental effects on neuronal survival, by creating a hostile and non-permissive environment for axonal repair. The transformation of astrocytes from reactive to scar-forming astrocytes highlights migration as a relevant regulator of glial scar formation, and further emphasizes the importance of efficient communication between astrocytes in order to orchestrate cell migration. The coordination between astrocytes occurs mainly through Connexin (Cx) channels, in the form of direct cell-cell contact (gap junctions, GJs) or contact between the extracellular matrix and the astrocytes (hemichannels, HCs). Reactive astrocytes increase the expression levels of several proteins involved in astrocyte migration, such as α_v_β_3_ Integrin, Syndecan-4 proteoglycan, the purinergic receptor P2X7, Pannexin1, and Cx43 HCs. Evidence has indicated that Cx43 HCs play a role in regulating astrocyte migration through the release of small molecules to the extracellular space, which then activate receptors in the same or adjacent cells to continue the signaling cascades required for astrocyte migration. In this review, we describe the communication of astrocytes through Cxs, the role of Cxs in inflammation and astrocyte migration, and discuss the molecular mechanisms that regulate Cx43 HCs, which may provide a therapeutic window of opportunity to control astrogliosis and the progression of neurodegenerative diseases.

## Introduction

Astrocytes are the most numerous glial cells in the central nervous system (CNS) and comprise nearly half the volume of the adult mammalian brain (Agulhon, 2008; Filous and Silver, 2016). As such, astrocytes are critical for supporting neuronal structure and brain homeostasis (Chung et al., 2015). Additionally, astrocyte functions include metabolic regulation of neurons, synaptic support, establishment of the blood–brain barrier (BBB), and a defense mechanism that constrains an injured or damaged site (Brown and Ransom, 2007; Sofroniew and Vinters, 2010; Pekny, 2016).

During development, differentiating newborn astrocytes undergo migration in order to reach their final destination (Goldman, 1997), whereas astrocytes in the adult brain are quiescent under normal physiological conditions. These star-like cells are arranged in the brain as tiling domains, where they do not intermingle their processes (Halassa, 2007; Cao, 2010). This segregation of processes is thought to occur by contact inhibition during postnatal development and is lost in disease or post-injury conditions (Sofroniew, 2009).

**Figure 1:**
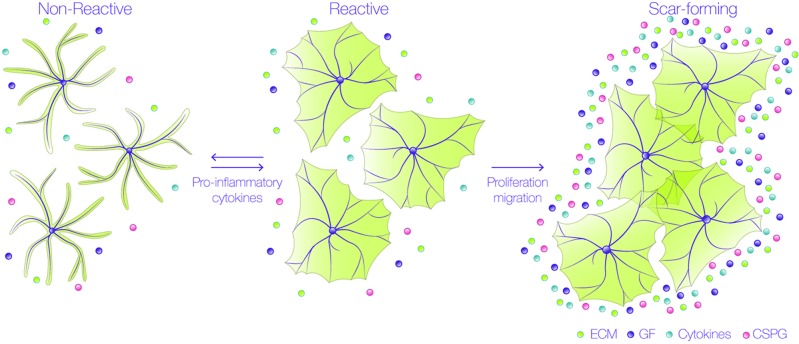
Astrocytes undergo astrogliosis in a pro-inflammatory environment. Astrocytes change their morphology from a non-reactive into a reactive state when exposed to pro-inflammatory cytokines.They undergo hypertrophy and not only change their shape, but also their protein expression; up to this stage, the process is reversible and reactive astrocytes do not overlap their branches. These cells then proliferate and migrate to the lesion site to form the glial scar, where they secrete many factors, such as extracellular matrix (ECM) proteins, cytokines, growth factors (GF), and chondroitin sulfate proteoglycans (CSPG). In this scar-forming stage, astrocytes no longer move, and the process is irreversible.

Events occurring in response to brain damage involve the participation of glial cells and, particularly, astrocytes. During the first stages of the lesion, damaged axons are exposed to inhibitory molecules, such as those found in the myelin sheath of oligodendrocytes. Interaction of neuronal receptors with these myelin ligands results in low regenerative capacity of the injured neuronal processes (Cao, 2010). Additionally, astrocytes undergo varying morphological and molecular changes after damage, through a process called reactive gliosis (**Figure 1**) (Sofroniew, 2009; Burda and Sofroniew, 2014), which is triggered by different molecules derived from the blood, inflammatory cells, or released from injured cells, such as adenosine trisphosphate (ATP), endothelin-1, and the pro-inflammatory cytokines tumor necrosis factor (TNF), interleukin-1β (IL-1γ), interferon gamma (IFNγ) and IL-6 (Giulian, 1988; Ahmed, 2000; John et al., 2003; Gadea et al., 2008). The response of astrocytes during gliosis varies according to their proximity to the injured site. Thus, astrocytes close to the injury change from a quiescent to a reactive state, in which astrocytes suffer cellular hypertrophy, acquire a fibroblast-like amoeboid morphology, and increase the expression of diverse proteins, such as glial fibrillary acidic protein (GFAP), vimentin, nestin, and the inducible nitric oxide synthase (iNOS) (Miyake, 1988; Clarke, 1994; Lagos-Cabre, 2017). After severe injury, there is a pronounced hypertrophy of the astrocyte cell body and processes, and astrocytes migrate to the injured site, where they increase their proliferation. These notorious changes significantly decrease individual astrocyte domains and therefore, the processes arising from several astrocytes overlap and form the glial scar, which isolates the damaged tissue and protects the adjacent nerve cells from harmful molecules (Homkajorn et al., 2010). The confinement of the damaged area after an injury requires that astrocytes polarize and migrate to the affected zone, where they avoid propagation of the lesion by the uptake of extracellular signals, such as glutamate, free iron, cytokines, ATP, ADP, or adenosine (Bylicky et al., 2018). Interestingly, these are the same molecules that induce the reactive phenotype in the first place. Therefore, reactive gliosis not only protects CNS cells from further damage, but also exerts harmful effects on neuronal survival and axonal regeneration (Pekny and Pekna, 2014; Sofroniew, 2014). Within 24 h after injury, and during the formation of the glial scar, astrocytes increase the secretion and deposition of chondroitin sulfate proteoglycans (CSPGs) into the extracellular matrix (ECM) which, together with the myelin-associated inhibitory molecules, create a hostile and non-permissive environment for axonal repair (Jones et al., 2003).

Reactive astrocytes can be classified as naïve (non-reactive), reactive, or scar-forming astrocytes, depending on their location and markers. Naïve and scar-forming astrocytes do not move, and astrocytes in the glial scar express N-Cadherin (Kanemaru, 2013). In contrast, reactive astrocytes (that move), express β-catenin, and metalloproteases, such as MMP2 and MMP13 (Verslegers, 2013). These important hallmarks suggest that there is a temporal sequence in the progression from naïve to reactive astrocytes, and then from reactive to scar-forming astrocytes. Since reactive astrocytes migrate to the injury site, isolate inflammatory cells and help repair tissue, this reactive stage constitutes a window of opportunity for interventions given that, up to this point, the process is reversible (**Figure 1**). These findings indicate that astrocyte migration is an important regulator of glial scar formation and highlight the relevance of studying the molecular mechanisms that regulate astrocyte motility. Additionally, in order for astrocytes to capture the signals of their surrounding microenvironment, they need to efficiently communicate with each other to orchestrate and synchronize, accordingly, each step of their movement. This coordination is achieved mainly by Connexin (Cx) channels, that can establish two distinct forms of communication: either through gap junctions (GJs), allowing direct cell-cell communication, or through hemichannels (HCs), that provide a pathway for the release and uptake of small molecules to and from extracellular compartments, respectively (Vicario, 2017). By sensing extracellular cues, astrocytes utilize their GJs or HCs in order to inform other cells of possible damage (Retamal, 2007). Furthermore, Cx HCs allow astrocytes to release molecules that can play a relevant role in autocrine/paracrine signaling in the brain (Retamal, 2007; Orellana et al., 2013; Alvarez, 2016; Lagos-Cabre, 2017), thereby potentiating important responses, such as cell migration (Alvarez, 2016; Lagos-Cabre, 2017).

The conversion of naïve astrocytes into motile and reactive cells observed after acute injury also occurs after stroke and neurodegenerative diseases, such as Alzheimer´s disease (AD) and Amyotrophic Lateral Sclerosis (ALS). Of note, reactive astrocytes up regulate the expression of several proteins that participate in astrocyte migration, such as α_v_β_3_ Integrin, the heparin sulfate proteoglycan Syndecan-4, the purinergic P2X7 receptor (P2X7R), as well as Cx43 and Pannexin1 (Px1) HCs (Lagos-Cabre, 2017).

Astrocytes are the cells with the highest level of Cxs in the CNS (Nagy and Rash, 2000). The first evidence of astrocytic Cxs that particularly formed GJs was obtained *in situ* by freeze-fracture electron microscopy (Brightman and Reese, 1969; Dermietzel, 1974). Later, in 1991, Cx43 was found to be one of the major Cx subtypes in astrocytes (Dermietzel, 1991). The pivotal role of Cxs in astroglial connectivity was demonstrated with Cx43/Cx30 double knockout (KO) mice, in which intercellular communication was lost (Dermietzel, 2000). However, the first relationship between Cxs and astrocyte migration was discovered in Cx43 KO mouse fetuses, using organotypic brain slice cultures that showed an irregular distribution of astrocytes (Perez Velazquez, 1996). Importantly, this finding led to the idea that Cx43 played a relevant role in regulating astrocytic mobility. Since then, several studies have reported that Cxs affect astrocyte migration (Homkajorn et al., 2010; Kotini and Mayor, 2015; Lagos-Cabre, 2018).

The focus of this review will be on the ability of Cxs to form HCs in astrocytes, in particular Cx43 HCs, and how they control astrocyte migration by releasing small molecules to the extracellular space. These molecules activate receptors in the same or adjacent cells, which then continue the signaling cascades required for astrocytes to move. We will also compare the functions of HCs and GJs in cell communication and the interplay between these two cellular channels in the regulation of cell migration.

## Astrocytes and Cell Communication

Astrocytes possess a characteristic star-like shape that distinguishes them from other non-neuronal cells of the glial family; however, despite the fact that astrocytes outnumber neurons and the other glia (i.e., microglia and oligodendrocytes) in rodents, their important role has always been undermined by neurons (Sosunov, 2014; Allen and Eroglu, 2017). In the human brain, there are many different types of astrocytes that can be identified by the combination of distinct cell markers, such as CD44, EAAT1, EAAT2, Aquaporin, and GFAP (Sosunov, 2014). The number of astrocytes in the human brain seems to vary according to the region, from 20–50%, and the exact ratio of total glial cells to neurons, although controversial, seems to be closer to one (von Bartheld et al., 2016).

The previous conception of astrocytes as being mere supporting cells for neurons is no longer valid. Today, it is known that astrocytes surround the pre- and post-synaptic membranes, thereby forming the “tripartite synapse” (Allen and Eroglu, 2017), and achieving functional integration and physical proximity to stimulate and regulate the activity of chemical synapses. Astrocytes also support and enhance the delivery of substrates required by neurons and act, for example, as a highway for glucose (Muller et al., 2018). Notably, and because astrocytes function primarily by anaerobic glycolysis, they can survive in low oxygen environments much longer than neurons. Astroglial Cx30 and Cx43 allow the diffusion of energy metabolites such as glucose and lactate and therefore, contribute to metabolic networks that are able to feed distant neurons in conditions such as hypoglycemia and/or high neuronal demand of energy substrates (Rouach, 2008). Astrocytes can also assist the metabolic needs of neurons by buffering molecules such as glutamate, K^+^, nitric oxide (NO), hydrogen peroxide (H_2_O_2_), and ammonia (Tsacopoulos and Magistretti, 1996; Dienel and Hertz, 2001; Aubert, 2007; Hertz et al., 2007). Astrocyte functions extend to the formation of the BBB by tightly apposing their end-feet to the endothelial cell vessels, thus helping with the maintenance of brain capillary permeability (Blanchette and Daneman, 2015; Zhao, 2015). In addition, astrocytes establish the principal defense mechanism after injury, surrounding the lesion site with their extended feet to avoid the propagation of damaging molecules (Ben Haim, 2015). To achieve all these functions, astrocytes need to sense and respond to signaling molecules, and then communicate with other astrocytes and their surroundings. Astrocytes display an extensive communication network by directly connecting cells through GJs, which are channels that consist of two facing connexons formed by a hexameric ring of Cxs, specifically Cx43/30 (Anders, 2014). Consequently, Cxs appear as one of the most important proteins related to cell communication in astrocytes, contributing to the coordination and maintenance of physiologic CNS function.

## Properties of Connexin Channels

**Figure 2:**
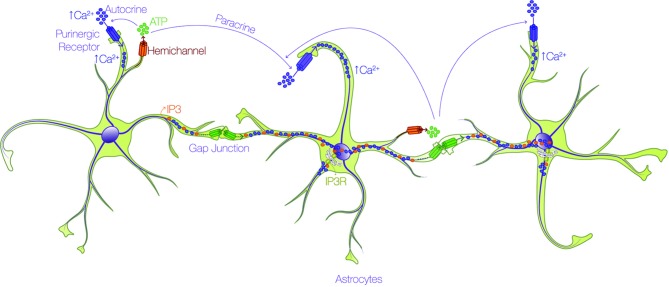
Astrocytes form an interconnected network through calcium. Astrocytes utilize the propagation of intercellular calcium (Ca^2+^) waves to achieve long-distance communication. There are two routes by which Ca^2+^ is mobilized through astrocytes: i) one pathway involves the passage of either IP_3_ (orange dots) or Ca^2+^ (purple dots) through gap junction channels (green connexon) and ii) the other route depends on the release of ATP (green dots) through hemichannels (orange connexon), and subsequent activation of purinergic receptors (purple pore) in the same (autocrine) or in neighboring (paracrine) cells, which promote Ca^2+^ uptake.

All Cxs share a similar topology, with four alpha-helical transmembrane domains connected by two extracellular loops and one intracellular loop, and two cytoplasmic N- and C-terminal domains (Bennett, 2016). The principal feature of Cxs is the capacity to form GJs for the interchange of metabolites and second messengers between contacting cells, or HCs that participate in paracrine and autocrine cellular signaling. HCs are permeable to different types of small molecules < 1.2 kDa, depending on the Cx isoform involved (Giaume, 2013; Oyamada et al., 2013; Nielsen, 2017; Nielsen, 2017): ions such as Ca^2+^, Na^+^, and K^+^; second messengers such as inositol 1,4,5 trisphosphate (IP_3_), cAMP, and cGMP; metabolites such as ATP, glutamate, glucose, and glutathione; and other small molecules (Kumar and Gilula, 1996; Kang, 2008; Bosch and Kielian, 2014; De Bock, 2014). This permeability allows the communication between cells through a complex syncytial network. The long-distance mechanism described in the early 90's for astrocyte communication *via* the intercellular passage of Ca^2+^ waves through GJs (Cornell-Bell, 1990) is debatable at present. The velocity of transport of IP_3_ through GJs for example, is 100-fold faster than that of Ca^2+^ itself (Allbritton et al., 1992; Hofer et al., 2002; De Bock, 2014), and because IP_3_ might release Ca^2+^ from intracellular stores by activating IP_3_ receptors (IP_3_R) (Allbritton et al., 1992; Hofer et al., 2002) rather than by directly moving Ca^2+^ as initially thought, the passage of IP_3_ molecules through GJ channels allows faster communication between cells. In the case of ATP released through HCs (Stout, 2002), accumulating evidence indicates that it activates purinergic receptors in the same (autocrine) or in neighboring (paracrine) cells, which induces the Ca^2+^ influx (Suadicani, 2004; Henriquez, 2011; Scemes and Spray, 2012; Alvarez, 2016; Lagos-Cabre, 2017) required for the propagation of Ca^2+^ (**Figure 2**).

The electrophysiological properties of Cxs are well known and their conductance allows to differentiate them and confirm the presence of specific Cxs in a given cell type (Retamal, 2007; Giaume, 2013). Cx HCs, also named connexons, can be formed by hexamers of the same (homomeric) or different (heteromeric) Cx subunits, while in the case of GJs, they are called homotypic or heterotypic when the two channels are formed by either homomeric or heteromeric connexons (Kumar and Gilula, 1996). On the other hand, the permeability of HCs and GJs also relies on subunit composition. For example, GJs formed by Cx40, Cx43 or Cx45 in cardiac cells show high permeability for several dyes with molecular weight above 400 Da, including Lucifer Yellow (LY) and propidium iodide (PI); in contrast, Cx30.2 shows no permeability for these two dyes (Rackauskas et al., 2007). The presence of Cx30.2, even in heterotypic GJs, precludes the permeability for LY or PI, suggesting that the presence of a non-permeable subunit is enough to completely modify the properties of GJs (Rackauskas et al., 2007). Interestingly, Cxs such as Cx30.2, which are permeable to small molecules, would be more adapted for electrical communication rather than metabolic transfer. Furthermore, GJs show selective permeability for biologically relevant molecules, such as second messengers; for instance, GJs formed by Cx43 have a 3-fold increase in permeability to cAMP compared to those formed by Cx26, and a 30-fold increase in permeability when compared to Cx36 channels, as tested in HeLa cells (Bedner, 2006). To add another level of complexity, Cxs can form homocellular GJs, such as neuron-neuron and astrocyte-astrocyte GJs, and heterocellular GJs, such as those formed between neurons and astrocytes (Nagy and Rash, 2000). Thus, the specific permeability properties and features of Cxs depend on the functionality of the distinct channels that they form. This specificity regulates channel conductance, electrical communication and metabolic coupling between cells (Vicario, 2017).

Since Cxs have a short life of only 1–5 h (Berthoud, 2004), the synthesis and delivery of new Cx proteins to the membrane is coupled to simultaneous GJ internalization, recycling to the membrane and Cx degradation (Segretain and Falk, 2004; Gilleron, 2009). Evidence has shown that Cxs can also be regulated by different types of post-translational modifications, like phosphorylation/dephosphorylation; and changes by oxidation, including effects of NO, hydrogen sulphide, or carbon monoxide, but not sulphur dioxide (Pogoda, 2016). Other modifications include acetylation, methylation, or ubiquitination (Pogoda, 2016). The stability of Cx43 on membranes depends, in part, on its interaction with the actin-associated proteins Zonula Occludens protein 1 (ZO-1) and Drebrin. The dissociation of Cx43/ZO-1 and Cx43/Drebrin from the cytoskeleton, through Src, has been found to promote Cx43 instability (Suh et al., 2012; Ambrosi, 2016; Sorgen, 2018).

Post-translational modification of Cxs is mainly represented by phosphorylation processes. Therefore, Cxs significantly interact with various protein kinases, as well as phosphatases. The cytoplasmic carboxy-terminal tail region of Cxs serves as a substrate for several kinases (Lampe and Lau, 2004; Marquez-Rosado, 2012), such as Cdk5 (Qi, 2016), ERK1/2 (De Vuyst, 2009), Akt (Park, 2007), PKA (Solan and Lampe, 2014), and PKC (Ek-Vitorin, 2006). The phosphorylation of Cx43 by Cdk5 on Ser279 and Ser282 decreases its membrane targeting and promotes its proteasomal degradation (Qi, 2016). GJs can be internalized after their ubiquitination as annular junctions in a clathrin-dependent process, and are sorted through the endosomal/lysosomal degradation pathway (Laird, 2006). In addition, Cx43 phosphorylation on S279/282 decreases GJ channel gating (Cottrell, 2003). On the other hand, Akt phosphorylates Cx43 in S373, forming larger GJs with higher communicational potential; this facilitates the turnover of GJs *via* the formation of an annular complex (Solan and Lampe, 2014). Moreover, Akt, PKA and PKC hierarchically phosphorylate Cx43 on various serine residues, thereby regulating the binding and release of ZO-1 from GJs, events that determine GJ function and endocytosis (Solan and Lampe, 2014; Thevenin, 2017).

Despite the large number of kinases that phosphorylate Cxs, HC activity is regulated only by PKA, PKC, MAPK and Akt (Pogoda, 2016). PKC-mediated phosphorylation of HCs formed by Cx43 abolishes sucrose and LY permeability by conformational changes in the structure of Cx43 (Bao et al., 2004). While phosphorylation by PKC closes Cx43 HCs, evidence from osteocyte cells indicates that their opening induced by shear stress depends on Cx43 phosphorylation by Akt on Ser369/Ser373 (Batra, 2014). Additionally, functional studies with lipid vesicles containing Cx43 HCs pre-loaded with fluorescent probes have indicated that phosphorylation of Cx43 by MAPK reduces the permeability of these liposomes (Kim, 1999). Given the large number of phosphorylation sites on Cx43, phosphatase-mediated dephosphorylation of Cx43 has been reported as an enhancer of HC permeability (Kim, 1999), while in GJs this post-translational modification enforces structural changes that reduce their functional coupling in astrocytes (Li et al., 2005). The role of serine/threonine phosphatases is to limit GJ conductance and enhance HC permeability. Thus, the regulation of Cx expression and activity has become a rich field of study for the analysis of their functional role in different physio-pathological conditions and today, GJs and HCs are not just viewed as mere connection proteins but rather as important regulators of cellular function.

## Astrocytes and Connexins During Inflammation

Different Cx isoforms are expressed in the brain. Thus far, 11 of the 21 Cx isoforms that have been described have been detected in the CNS (Mayorquin, 2018). Different types of astrocytes express several Cxs (Beyer, 2001; Giaume, 2013; Mansour, 2013; Bosch and Kielian, 2014), with Cx 30 and Cx43 being the mayor ones (Giaume and McCarthy, 1996). Additionally, Cx26, Cx30, Cx40, Cx45, and Cx46 mRNA has been detected in cultured astrocytes from Cx43 KO mice (Dermietzel, 2000), mRNA for Cx26, Cx30, Cx32, Cx40, and Cx43 has also been detected by single-cell RT-PCR in hippocampal astrocytes (Blomstrand, 2004), and GJs in cultured astrocytes are mainly composed of Cx43 (Dermietzel, 1991; Giaume, 1991). Cxs in astrocytes, oligodendrocytes, microglia and neurons are characterized according to the developmental state, region and cell-type specific isoform expression, suggesting that Cxs play a critical role in the regulation and maintenance of various CNS functions (Lapato and Tiwari-Woodruff, 2018). Cx43 is ubiquitously expressed in astrocytes throughout the brain, and along with Cx26 and Cx30, contributes to the interconnection of the astrocyte network (Rash, 2001); however, Cx26 and Cx30 are less abundant in astrocytes (Contreras, 2004). This expression profile probably determines the autocrine and paracrine signaling interaction that mediates glial and neuroglial communication (Lapato and Tiwari-Woodruff, 2018). Importantly, Cx43 is upregulated under inflammatory conditions and in astrocytes derived from transgenic hSOD^G93A^ mice, which is an animal model of ALS. The astrocytes of ALS mice exhibit increased number of GJs, active HCs, and elevated levels of intracellular Ca^2+^ concentration ([Ca^2+^]_i_) (Almad, 2016; Lagos-Cabre, 2017). Additionally, pharmacological blockade of Cx43 with both GJ or HC blockers offers neuroprotection to motor neurons cultured with hSOD^G93A^ astrocytes, suggesting a detrimental role of Cx43 in ALS neurodegenerative models (Almad, 2016). Blocking Cx43 has also shown protective effects in other neurodegenerative conditions, such as hypoxia and glaucoma (Vicario, 2017). Moreover, strategies combining Cx mimetic peptides to target glial and endothelial GJs and HCs with drugs that preclude electrical synaptic signaling pathways have been considered to improve survival of neurons in neurodegenerative diseases and injuries. These mimetic peptides have revealed a reduction in inflammatory signaling after blockage of Cx43 HC activity (Moore and O'Brien, 2015).

Given the extensive expression and regulation of Cxs in glial cells, there has been a significant interest in the role that they play in different neuropathologies. These diseases are not only specific to the CNS, but also involve the peripheral nervous system, among other systems (Abrams and Scherer, 2012). A number of these brain pathologies are associated with glial reactivity, and since Cx43 is highly expressed and regulated in astrocytes, relevant correlations of Cx43 changes are related with these pathologies (Giaume, 2013). Both in human tissue as well as in animal models, changes in Cx43 expression have been associated with ischemia and stroke, epilepsy, brain infection, inflammation and traumatic brain injury (Giaume, 2010). Furthermore, Cx43 also plays a relevant role in neurodegenerative diseases such as AD, Parkinson´s disease, ALS, Multiple sclerosis (Xing, 2019) and neuropsychiatric diseases, including major depressive disorder (Kim, 2018), highlighting the deleterious effect of compromising Cx43 functions in astrocytes.

On the other hand, we have reported in non-reactive astrocytes, that β_3_ Integrin overexpression leads to increased Cx43 levels (Lagos-Cabre, 2017), suggesting that Cx43 regulatory elements are downstream of β_3_ Integrin-induced signaling. This agrees with reports showing that β_3_ Integrin can regulate the transcription factor NF-κB, which in turn, would regulate Cx43 expression by binding to its promoter (Alonso, 2010; Balasubramaniyan, 2013). Reports indicate that Cx expression is controlled by several common and well known transcription factors, such as Sp1, Sp3 and AP-1 (Oyamada et al., 2013). However, tissue-specific expression of Cxs is regulated by particular transcription factors, such as NKx2.5, Shox2, or Tbx5 for cardiac tissue Cxs; HNF1 and Mist for digestive system Cxs, and Wnt or Sox10 for neural tissue-related Cxs (Oyamada et al., 2013). In astrocytes, ciliary neurotrophic factor receptor α (CNTFRα) appears as a regulator of Cx43 expression by binding to CNTF-response elements (Ozog, 2004; Oyamada et al., 2013). Importantly, as previously mentioned, β_3_ Integrin is upregulated under inflammatory conditions in the brain (Lagos-Cabre, 2017) and therefore, a clear link between Cx43 and the β_3_ Integrin seems to exist in disease progression.

Pannexins (Pxs) are proteins similar to Cxs, but only structurally related and without sequence homology (Panchin, 2000). This protein family is composed of three members (Px1, Px2 and Px3), which are orthologues to insect innexins (Panchin, 2000; Baranova, 2004; Giaume, 2013). Despite the capacity of innexins to form GJs in insects, Pxs appear to form only HCs in mammals (Giaume, 2013). However, Pxs can form GJs when they are overexpressed in mammalian cells (Vanden Abeele, 2006). Px1 is the most studied and most ubiquitous Px. Px2 has been related to neuronal differentiation and tumor development processes, while Px3 has been involved in osteoblast and chondrocyte differentiation and sperm transportation (Bruzzone, 2003; Baranova, 2004; Turmel, 2011; Penuela et al., 2013). Interestingly, astrocytes express Px1 and Px2 (Giaume, 2013), and our own work indicates that Px1 is upregulated in astrocytes treated with the pro-inflammatory cytokine TNF or in astrocytes that overexpress β_3_ Integrin (Lagos-Cabre, 2017). Therefore, an interesting possibility is that Px1, as observed for innexins, might form GJs in reactive astrocytes, in which Px1 is upregulated. In addition, Px1 participates, together with Cx43, in astrocyte migration induced by neuronal cues (Alvarez, 2016). Intriguingly, functional Px1 channels have been found in several blood components, such as red blood cells and platelets (Isakson, 2017). However, red blood cells lack Cx43 and do not promote vesicular release of ATP under physiological conditions (Locovei et al., 2006; Qiu, 2011); thus, the dynamic flow of red blood cells, which depends on the ATP released from the intracellular space, occurs through Px1 rather than Cx43 channels (Forsyth, 2011).

Astrocyte reactivity is a response to any pathological condition in the CNS, characterized not only by reactive gliosis, but also by the activation of mononuclear phagocytes, neuronal injury, and cell death, events which normally are linked to changes in the activity and regulation of several major CNS Cxs, such as Cx29, Cx30, Cx32, Cx36, Cx43, and Cx47 (Decrock, 2015; Belousov, 2017). Reactivity in astrocytes not only manifests with changes in cell morphology, but also at the level of expression and activity profile of various proteins, including Cxs and Pxs (Retamal, 2007; Homkajorn et al., 2010; Giaume, 2013; Bosch and Kielian, 2014; Ben Haim, 2015; Abudara, 2015; Alvarez, 2016; Almad, 2016; Garré, 2016; Grygorowicz et al., 2016; Lagos-Cabre, 2017; Yi et al., 2017). Interestingly, at least in the case of ALS and the animal model of multiple sclerosis (experimental autoimmune encephalomyelitis, EAE), it seems that the reactive phenotype in astrocytes is achieved at early stages of the disease, even before the appearance of early symptoms (Levine, 1999; Grygorowicz et al., 2016). In the ALS mouse model, for example, astrocytes derived from the spinal cord of neonatal mice show reactive phenotype markers after 14 days of *in vitro* culture (Lagos-Cabre, 2017). Considering that in this animal model, the symptoms only appear after 3 months (Gurney, 1994; Rojas, 2014), reactive astrocytes may play an important role in the onset and progression of this neurodegenerative disease. Likewise, early appearance of astrogliosis markers has been recently reported in an induced EAE rat model (Grygorowicz et al., 2016). In this study, the authors show that as early as 2-4 days post induction of EAE, the levels of GFAP and S100β (another gliosis marker) are elevated, whereas the first symptoms manifest only after 10 days post EAE induction (Grygorowicz et al., 2016). These findings suggest that astrocyte reactivity is an early, if not the first step, in the onset of these diseases.

Intriguingly, the reactive phenotype is also achieved *in vitro* by the addition of pro-inflammatory cytokines such as IL-1β and TNF, or by the addition of conditioned medium from activated microglia (Retamal, 2007; Lagos-Cabre, 2017), suggesting that astrocytes in culture retain all the relevant components that can trigger the reactive response. Pro-inflammatory molecules not only upregulate astrocyte Cx43 and Px1, but also increase β_3_ Integrin expression levels and induce astrocyte reactivity (Lagos-Cabre, 2017). Moreover, the reactive phenotype in astrocytes can also be achieved by overexpression of proteins in the absence of cytokine treatments. We have recently reported that by overexpressing β_3_ Integrin, astrocytes increase the expression of reactivity markers, such as GFAP and iNOS, and attain a functional reactive phenotype by increasing Cx43, Px1, and P2X7R expression levels and ATP release. These changes make astrocytes responsive to external cues that promote cell polarization and migration (Lagos-Cabre, 2017; Lagos-Cabre, 2018). On the other hand, silencing of β_3_ Integrin precludes stimulus-induced astrocyte migration even when the cells are treated with TNF (Lagos-Cabre, 2017). Additionally, Strużyńska's group described a temporally coincident elevated expression of Cx43, P2X7R and reactivity markers, where the sole blockade of P2X7R decreased astrogliosis and ameliorated EAE symptoms in an animal model (Grygorowicz et al., 2016). In the same line, Cx43 mimetic peptides have been reported to reduce astrogliosis and cytokine release, improving function after spinal cord injury (O'Carroll, 2013). These results, together with the recent findings that support the reversibility of astrocyte reactivity (Hara, 2017), indicate that the regulation of the signaling pathway that involves HC opening, ATP release, and the activation of the P2X7R might provide a therapeutic window of opportunity to control astrogliosis and the progression of neurodegenerative diseases.

Despite the capacity of Cxs to form GJs, HCs formed by these proteins seem to be mostly affected by a pro-inflammatory environment. For example, the strong reactivity of astrocytes observed in AD is accompanied by an increase in the activity of Cx43 HCs, which maintain the reactive phenotype by releasing toxic molecules to the extracellular space (Yi et al., 2017). In pilocarpine-induced status epilepticus mice, Cx43 and Cx40 levels increase in GFAP-positive astrocytes, effect that lasts for at least 2 months in the hippocampus (Wu, 2015). Similarly, in astrocytes treated with conditioned media from microglia activated by LPS, Giaume and co-workers found an increase in astrocyte permeability, along with a decrease in GJ communication (Retamal, 2007), demonstrating the importance of HCs -rather than GJs- during inflammation.

As stated above, increased levels of Cx43 during astrocyte reactivity help maintain the reactive phenotype of astrocytes and microglia by releasing ATP, glutamate and other molecules to the extracellular space, generating a positive feedback loop (Ben Haim, 2015). In the same context, high Cx43 levels in reactive astrocytes derived from ALS mice help sustain an increase in [Ca^2+^]_i_ induced by mechanical or ATP stimulation, which is abolished by a Cx43-blocking peptide (Almad, 2016). In agreement with these findings, mice with genetically reduced levels of Cx43 show attenuation of LPS-induced sepsis, which includes reduction of activated microglia and cytokine production (Zhou, 2015). These reports highlight Cx43 as a key element to maintain the astrocyte reactive phenotype by promoting ATP release and Ca^2+^ signals.

**Figure 3:**
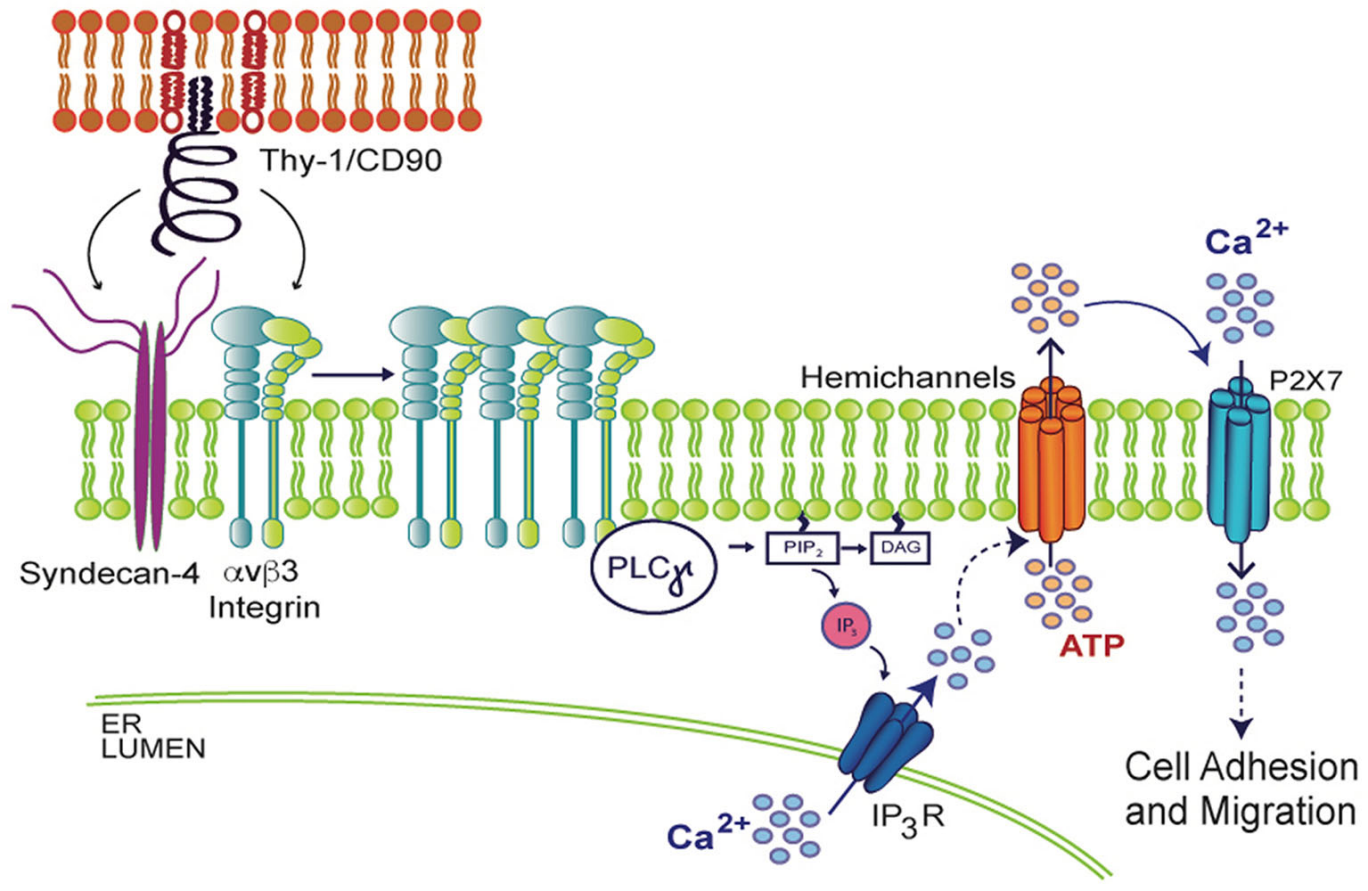
Molecular mechanism involved in Thy-1/CD90-induced astrocyte adhesion and migration. In the context of neuron (upper red lipid bilayer) and astrocyte (lower green lipid bilayer) communication, neuronal Thy-1/CD90 interacts with both α_v_β_3_ Integrin and Syndecan-4 astrocytic receptors, triggering PLCγ activation, IP_3_ production, IP_3_R activation, increase in cytosolic Ca^2+^, and opening of hemichannels and subsequent ATP release. Extracellular ATP mediates P2X7R integrin-dependent transactivation, allowing Ca^2+^ entry, which results in morphological changes of astrocytes (increased adhesion) and later, cell migration.

When spinal cord astrocytes are stimulated with fibroblast growth factor 1 (FGF-1), which stimulates astrocyte reactivity as well, increased Px1 and Cx43 HC opening induces cell permeability, ATP release and [Ca^2+^]_i_ increase (Garré, 2016). Interestingly, the opening of these HCs is prevented by the addition of a Phospholipase C gamma (PLCγ) inhibitor or by loading cells with BAPTA-AM (Garré, 2016), suggesting that Ca^2+^ signals likely derived from activation of IP_3_R in the endoplasmic reticulum (ER) are involved in HC opening. Supporting this idea, *in vivo* studies have shown that after a brain cortex injury, the surrounding astrocytes become reactive. Interestingly, their reactivity can be prevented with BAPTA-AM, which reduces GFAP levels and glial scar formation (Gao, 2013), demonstrating the requirement of Ca^2+^ signals in this process. Similarly, in the astrocyte DITNC1 cell line, as well as in primary astrocytes treated with TNF, Ca^2+^ is released from the ER and ATP is released through HCs, in a complex signal transduction cascade that results in changes in cell shape and initiation of cell migration when stimulated with the neuronal protein Thy-1/CD90 (Henriquez, 2011; Alvarez, 2016; Lagos-Cabre, 2017; Lagos-Cabre, 2018). Thy-1/CD90 is a glycoprotein from the neuronal surface that binds to astrocytes by engaging α_v_β_3_ Integrin and Syndecan-4 receptors, recruiting diverse focal adhesion proteins that include PLCγ. The activation of PLCγ results in DAG and IP_3_ production and consequent IP_3_R activation, Ca^2+^ release from the ER, and opening of Cx43 and Px1 HCs, which release ATP to the extracellular space. ATP then binds to the P2X7R, allowing Ca^2+^ entry and thus, inducing morphological changes and cell migration (Henriquez, 2011; Alvarez, 2016; Lagos-Cabre, 2017; Lagos-Cabre, 2018) (**Figure 3**). These findings demonstrate the ability of HCs to release molecules that sustain an increased [Ca^2+^]_i_ to maintain the astrocyte reactive phenotype and therefore, suggest that Ca^2+^ is a key player in the modulation of astrocyte reactivity. Since migration of astrocytes under either physiological or pathological conditions is a very complex process, future systematic studies are needed to fully elucidate the relevant role of Ca^2+^ in astrocyte migration and reactivity.

## Regulation of Cell Migration by Connexins

Cell migration is an essential process for the development, maintenance and healing of multicellular organisms. By sensing their environment, cells polarize, extend filopodia and lamellipodia to the leading front, adhere to the ECM proteins through integrins and Syndecan-4, and form focal adhesions and bundles of actin microfilaments called stress fibers. These focal points of adhesion to ECM proteins, along with stress fibers, allow cells to contract their rear and promote forward cell movement (Ladoux and Mege, 2017). Until now, astrocyte migration has not been studied extensively and detailed mechanisms remain largely unknown. While single-cell migration has been studied in depth, collective cell migration is a less studied process that refers to the coordinated movement of cell groups, sheets, or chains (De Pascalis and Etienne-Manneville, 2017). However, collective cell migration cannot be simplified as a group of independent cells that move at the same speed and direction; but as a more complex phenomenon that can improve migration efficiency by rendering cells with specific features (Mayor and Etienne-Manneville, 2016). Just as single cells, migrating cell groups are equally relevant; they govern collective cell migration during embryonic development, wound healing, and cancer cell invasion, among other processes (Ladoux and Mege, 2017). Collective cell migration relies on both cell-environment, as well as cell-cell interactions, and on several proteins related to cell-cell communication, including proteins forming not only GJs, but also adherens junctions and tight junctions (Ladoux and Mege, 2017).

Cxs participate in the migration of astrocytes (Homkajorn et al., 2010; Alvarez, 2016; Lagos-Cabre, 2017) and several other cell types, such as neurons (Qi, 2016; Laguesse, 2017), cancer cells (Graeber and Hulser, 1998), keratinocytes (Jaraiz-Rodriguez, 2017) and bone marrow stromal cells (Jin, 2017). Reports have indicated that the mutant Cx26^S17F^, related to keratitis-ichthyosis-deafness syndrome (KIDS), reduces GJ communication, and decreases collective migration of primary keratinocytes (Press, 2017). Interestingly, despite the fact that Cx26^S17F^ mice show normal skin wound closure, their repaired zone is thicker than in controls, suggesting abnormal remodeling (Press, 2017). Similarly, wound-healing assays with HeLa cells that overexpress Cx26 show increased Rac1-dependent cell migration, along with downregulation of N-Cadherin (Polusani, 2016). It appears that the reduced levels of N-Cadherin release a break for cell migration that acts by “sequestering” Rac1 and other cellular components near the membrane; thus, when N-Cadherin levels go down, Rac1 is released and activated, allowing cell migration (Polusani, 2016). Of note, these authors also show that decreased levels of both N-Cadherin and cell migration are dependent on Cx26-forming GJs, but not HCs (Polusani, 2016). Therefore, GJs seem important for the regulation of collective cell migration, in processes such as skin wound repair and tumor invasion.

On the other hand, Cx43 favors migration of projection neurons over radial glial cells in the developing brain (Laguesse, 2017). In this report, it is indicated that Cx43 favors cell-to-cell contact by interacting with elongator complex elements such as Elp1 and Elp3, allowing the acetylation of Cx43 and its membrane localization (Laguesse, 2017). Such membrane destination of Cx43 dependent on acetylation levels has also been reported in HeLa cells (Laguesse, 2017). An important observation in these studies is that channel activity was not required for neuronal migration. In other cases, the function of GJs as channels seems less clear, but cellular localization of Cx43 at the plasma membrane also seems to control cell migration by favoring cell adhesion. It will be important to determine if these “channel-dependent” or “channel-independent” functions require the presence of functional GJs or HCs, respectively, and whether or not these Cx structures acting as scaffolds are also important for cell migration (Kameritsch et al., 2012).

Accumulating evidence has also indicated that Cxs can enhance and inhibit cancer cell migration, depending on the stage of the disease and tissue involved (Kotini and Mayor, 2015). Cx26 and Cx43 expression levels are increased in invasive lesions and in lymph node metastases of breast cancer (Jamieson, 1998; Kanczuga-Koda, 2006). Overexpression of Cx43 in breast cancer metastatic cell lines enhances tumorigenesis without affecting GJ formation or cell motility (Li, 2008). Another report has indicated a correlation between Cx43 levels and metastatic potential in prostate cancer cells (Zhang, 2015), whereas in testicular cancer cells resistant to cisplatin, overexpression of Cx43 reduces migration/invasion of these cells (Wu, 2018). More importantly, the role of Cx43 in cell migration was first described in breast MCF-10A epithelial cells using a siRNA screening approach designed to identify genes that regulate cell motility (Simpson, 2008). In these cells, Cx43 controls migration and directionality, since knockdown of Cx43 leads to erratic, slow and reverse migration. This could be related to the increased capacity of MCF-10A cells to form protrusions, which results in cells with a more polygonal shape and diminished ability to migrate. Interestingly, a similar cellular shape has been observed in cardiac neural crest cells from Cx43^-/-^ mice (Xu, 2006; Matsuuchi and Naus, 2013). Stachowiak and co-workers have shown that reincorporation of Cx43 through microvesicles derived from HeLa cells decreases migration of MDA-MB231 breast tumour cells (Ferrati, 2017). These Cx43-containing microvesicles are described to form GJs in these breast cancer cells, favoring the idea that functional GJs, rather than HCs, decrease cell migration. Considering these results, the role of Cx43 in cell migration still seems controversial. Perhaps, there is a critical amount of Cx43 at the plasma membrane that favors GJ formation, which might also determine the cellular ability to either move or remain stationary.

Accordingly, Cx43 has been involved in the inhibition of glioma cell migration (Jaraiz-Rodriguez, 2017). However, this effect relies on the interaction of Cx43 with c-Scr, and not on its activity as a channel or HC. In many cells, active c-Src phosphorylates and activates focal adhesion kinase (FAK), creating additional binding sites for protein-complex formation. These complexes induce formation of focal adhesions, which are essential for cells to adhere to a substrate and migrate (Dubash, 2009). Cx43 forms a complex with c-Src and inhibits Src activity by recruiting its inhibitor, C-terminal Src kinase (Csk), to the complex (Gonzalez-Sanchez, 2016). Therefore, Cx43 HCs could induce or repress cell motility by interacting with a different set of molecules, at least, in deregulated cells such as glioma cells and other cancer cells, where GJs can act as inhibitors of cell migration. Therefore, it seems clear that Cxs play an important role in cell migration in various cell types, but the final outcome is either membrane expression level- or cell-context-dependent.

In summary, despite available information concerning the mechanisms governing cell migration in various cell types, astrocyte migration still requires future research in order to better understand the molecular mechanisms that Cxs use to regulate motility, in order to serve as potential targets for the development of clinical interventions for astrogliosis and glioma metastasis.

## Connexins and Astrocyte Migration

Astrocytes in the adult brain are non-migratory cells; *i.e.*, are quiescent under normal physiological conditions. However, they can be activated to become migratory under pathological conditions such as trauma, ischemia, infection, inflammation and neurodegeneration (Zhan, 2017). Recent *in vivo* studies indicate that reactive astrocytes undergo hypertrophy, cell polarization, and cell migration (Bardehle, 2013; Moore and Jessberger, 2013; Sirko, 2013). Conversely, astrocytes reportedly undertake migration upon injury or other pro-inflammatory conditions to form a glial scar and repair the area of the lesion (Bush, 1999; Faulkner, 2004; Sofroniew, 2005; Chai, 2013). Results from embryonic brain slices of Cx43 KO mice show abnormal distribution of astrocytes when compared with the normal counterpart (Perez Velazquez, 1996; Kotini and Mayor, 2015). Similar experiments performed in a subline of Cx43 KO mice called “Shuffler”, which exhibits defects in brain architecture and astrocyte distribution, strongly suggest migration defects of astrocytes lacking Cx43 (Wiencken-Barger, 2007; Kotini and Mayor, 2015). Our own findings with neonatal rat astrocytes activated *in vitro* by the addition of TNF or other cytokines, indicate that only reactive astrocytes move in response to external stimuli (Lagos-Cabre, 2017). In this context, Cx43 appears to be the most relevant HC-forming protein involved in reactive astrocyte migration, since the specific inhibitory peptide Gap19 abolishes HC opening and cell migration induced by neuronal Thy-1/CD90 (Alvarez, 2016; Lagos-Cabre, 2017). Therefore, pro-inflammatory signals that trigger astrocyte reactivity seem to be necessary for these cells to move in response to extracellular cues, and their migration is related to the presence of Cx43.

Thy-1/CD90 activates its two receptors, α_v_β_3_ Integrin and Syndecan-4, only in TNF-treated astrocytes (Leyton, 2001; Kong, 2013; Alvarez, 2016; Lagos-Cabre, 2017), likely because the expression levels of both receptors are enhanced upon pro-inflammatory conditions (Lagos-Cabre, 2017). Importantly, proteins upregulated by TNF treatment also include: Cx43, Px1, P2X7R, GFAP, and iNOS (Lagos-Cabre, 2017). The engagement of α_v_β_3_ Integrin and Syndecan-4 by Thy-1/CD90 in reactive astrocytes triggers similar intracellular signaling pathways as those described for DITNC1 astrocytes (see **Figure 3**), including Ca^2+^ release from the ER, opening of Cx43 and Px1 HCs, ATP release, and P2X7R activation, with the consequent further increase in [Ca^2+^]_i_ required for cell migration (Abudara, 2015; Alvarez, 2016; Garré, 2016; Grygorowicz et al., 2016; Lagos-Cabre, 2017). However, this molecular mechanism seems to be necessary only for mature astrocytes, since the addition of conditioned media from microglia or IL-1β to astrocyte progenitor cultures reduces cell migration and spontaneous Ca^2+^ oscillations in these cells (Striedinger and Scemes, 2008). These astrocyte progenitors also show release of ATP to the extracellular medium, but in an exocytosis-dependent fashion that also depends on Ca^2+^ (Striedinger et al., 2007).

Despite the key role of ATP in astrocyte migration, the addition of different concentrations of extracellular ATP to non-reactive astrocytes only induces a graded reactive phenotype, including proliferation and stellation; however, under these conditions, astrocyte phenotype is not accompanied by an increase in GFAP and cells do not migrate in wound-healing assays (Adzic, 2017). These results indicate that even though ATP triggers various attributes of activated astrocytes, this is not sufficient to induce a full reactive phenotype in astrocytes. On the contrary, Wang and coworkers showed that astrocytes migrate after ATP or UTP treatment and increase their GFAP and α_v_β_3_/β_5_ Integrin levels, of which the latter is important for astrocyte migration after UTP treatment (Wang, 2005). In this study, the authors utilize primary astrocytes in culture, and suggest that they migrate because the nucleotides induce astrocyte reactivity, which is supported by the increased expression of GFAP and integrins. Despite the fact that Cxs were not investigated in these studies, by adding ATP (or UTP) to the extracellular medium of astrocytes and inducing astrocyte reactivity, Cx43 may also be upregulated (Lagos-Cabre, 2017), possibly explaining the effect observed in cell migration.

**Figure 4:**
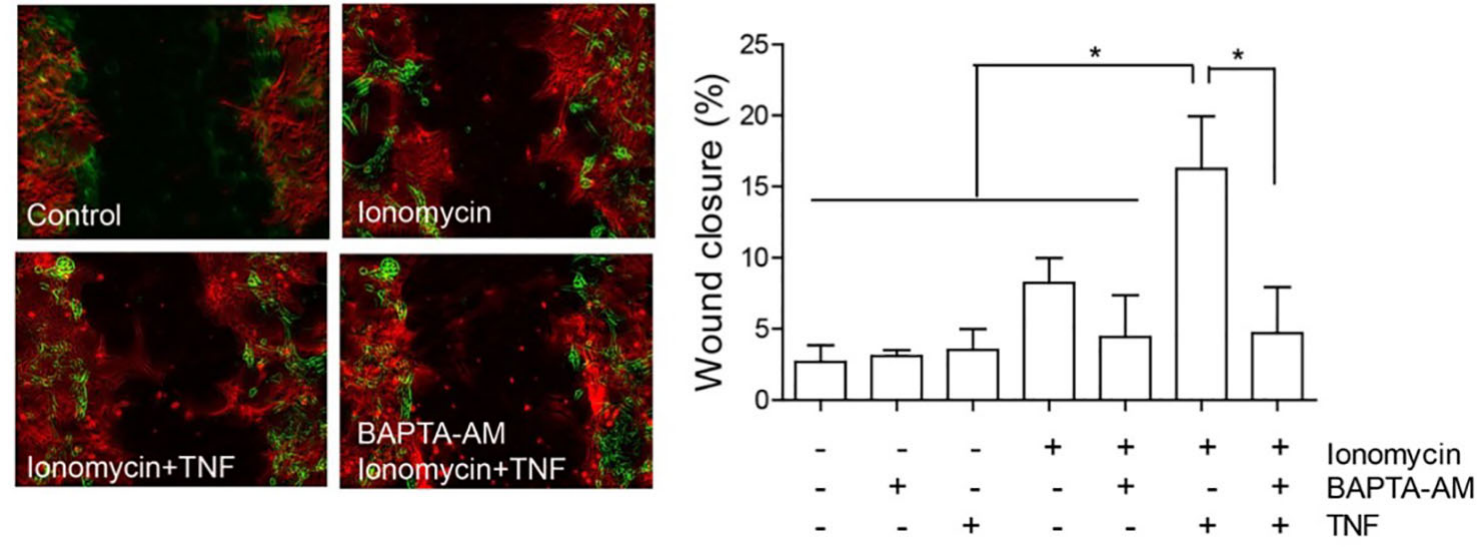
Astrocyte migration induced by ionomycin. Primary astrocytes from rat cortexes were isolated and cultured as published before (Lagos-Cabre, 2017). Astrocytes were seeded in 24 well-plates and treated or not with 10 ng/ml of TNF for 48 h. Astrocytes were then subjected to a scratch with a pipette tip and floating cells were washed away before treatment addition. Left panel: Representative images of the wound-healing assay with pseudocolor of selected treatments. The green color represents cells in the wound edge at 0 h and the red color, cells of the same wound 24 h after treatment. Right panel: Wound-healing assay quantification of astrocyte migration 24 h after treating cells with 1 µM of ionomycin. Where indicated, cells were pre-incubated with 5 µM BAPTA-AM for 30 min, prior to ionomycin addition. Wound closure was higher in cells treated with ionomycin + TNF, revealing increase of migration. Values in the graph represent mean ± s.e.m. of three independent experiments. The results were analyzed using one-way ANOVA and Tukey's post-test. Statistical significance is indicated, *p < 0.05.

The localization of Cx43 is also modified in reactive astrocytes. Under normal conditions, Cx43 is mostly localized in intracellular vesicles, but after the addition of TNF, it localizes in a near-to-membrane zone (Lagos-Cabre, 2017); this result also supports the importance of HCs in astrocyte reactivity and migration. Indeed, the levels of Cx43 at the plasma membrane could regulate ATP release and, as a consequence, increase [Ca^2+^]_i,_ which is necessary for cell migration (Alvarez, 2016; Lagos-Cabre, 2017). Consequently, any increase in [Ca^2+^]_i_ should lead to cell migration. In support of this assumption, Hayashi and coworkers observed that the increase in Ca^2+^ induced by ionomycin was necessary and sufficient to induce cell migration of leading edge mesodermal cells treated with this ionophore (Hayashi et al., 2018). Interestingly, our own results show that only partial cell migration (8.2 ± 1.8% wound closure compared to 2.7 ± 1.2% of control samples, **Figure 4**) is observed when astrocytes are treated with ionomycin in a wound healing assay, while after pre-treating with TNF, ionomycin significantly enhances migration (15.3 ± 4.1%), with respect to treatment with only ionomycin (**Figure 4**). Pre-incubation with BAPTA-AM completely abolishes astrocyte migration induced by ionomycin/TNF treatment (**Figure 4**), indicating the necessity of cytosolic Ca^2+^ for the response. However, although TNF alone does not produce changes in either [Ca^2+^]_i_, ATP release, or cell migration (Sofroniew, 2014), it appears to prime the cells to respond to additional stimuli. Considering that TNF induces astrogliosis, these results suggest that the reactive phenotype is a key step for astrocytes to move and that [Ca^2+^]_i_ increase, although necessary, is not sufficient to induce astrocyte migration. This potentiation of the migratory effect induced by TNF is interesting and suggests that the increase in P2X7R protein levels (Lagos-Cabre, 2017) (or other Ca^2+^ channels) could explain the difference in migration by further increasing the Ca^2+^ influx induced by ionomycin. Alternatively, since astrocyte migration involves elevation of cytosolic Ca^2+^ *via* both ER release downstream of integrin activation and uptake of extracellular sources through ATP-gated-P2X7R pores (**Figure 3**), it is possible that Ca^2+^ is elevated at specific times and places. Therefore, the bulk of Ca^2+^ induction by ionomycin does not mimic all events that are triggered by physiological ligands, such as Thy-1/CD90. We have previously shown that astrocytes require TNF to respond to Thy-1/CD90, which induces a robust elevation of [Ca^2+^]_i_ by the release of ATP and activation of the P2X7R (Lagos-Cabre, 2017). Here, we confirmed that an increase in [Ca^2+^]_i_ alone only slightly affects astrocyte migration, an event that further requires the molecules that are overexpressed by TNF treatment (such as Cx HCs) to maintain, for example, a reactive phenotype, or to sustain a positive feedback loop between ATP release, P2X7R activation, and [Ca^2+^]_i_ increase. In our previous reports we have shown that two Ca^2+^ sources are needed to induce astrocyte migration: one dependent on Ca^2+^ released from internal stores and triggered by integrin engagement, which is necessary for Cx43 HC opening; and another related to ATP release and P2X7R activation (Henriquez, 2011; Alvarez, 2016; Lagos-Cabre, 2017). Thus, although ionomycin increases [Ca^2+^]_i_ in an artificial manner, low levels of Cx43 and P2X7R at the plasma membrane -due to the lack of pro-inflammatory signals- could explain the reduced effect of the ionophore on cell migration.

Alternatively, enhanced [Ca^2+^]_i_ induced by ionomycin might stimulate the opening of different pores, other than Cx HCs, but that share P2X7R properties, as has been reported in 2BH4 thymic epithelial cells and peritoneal macrophages (Faria, 2009). The nature of this pore was not determined and the authors indicated that pore activation induced by [Ca^2+^]_i_ depends on calmodulin, PLC, MAPK, and cytoskeleton components (Faria, 2009). According to the scratch assay results that we show here, primary astrocytes treated with ionomycin alone increase their migration, but to a level not as high as that in cells pre-treated with TNF (**Figure 4**). Thus, the key event seems to be a pro-inflammatory stimulus that, apart from provoking elevated levels of many surface proteins (Lagos-Cabre, 2017), could regulate distinct intracellular signaling pathways that might activate the alternative pore proposed by Farias and coworkers.

We have proposed that TNF elevates β_3_ Integrin cluster formation in astrocytes by increasing the expression of α_v_β_3_ Integrin at the plasma membrane. A low level of clustering could trigger signaling cascades involved in focal adhesion formation, including PLCγ activation and Ca^2+^ release *via* IP_3_R activation to yet undetectable levels, but that prompts cells to quickly respond to stimuli like ionomycin. We have tested Cx43 HC opening by LY uptake in ionomycin-treated astrocytes and found that these HCs open even in the absence of TNF (Lagos-Cabre, 2018). These results suggest that: i) HC opening and astrocyte migration are two independent processes that can potentiate each other with Thy-1/CD90 stimulation (see above); ii) HC opening is only part of the mechanism required to be activated in order to trigger a response; and iii) the LY dye could be passing through a pore that is different from HCs, but that opens with ionomycin treatment (Faria, 2009). Additionally, the combination of ionomycin and Thy-1/CD90 induces lower migration levels than Thy-1/CD90 and TNF applied together in a Boyden chamber transmigration assay (Lagos-Cabre, 2018). The latter reinforces the idea that Thy-1/CD90 has a limited capacity to stimulate non-reactive cells and that ionomycin does not produce all the changes induced by TNF. Thus, astrocyte migration requires many molecular components increased by TNF and cannot be replaced by an artificial [Ca^2+^]_i_ increase, supporting the idea that Ca^2+^ is necessary, but not sufficient for astrocytes to migrate. Of note, the different elements that interact with each other to regulate astrocyte migration are also regulated by different signaling pathways related to various astrocytic functions. These mechanisms will provide insights for future research on astrocyte migration.

## Concluding Remarks

In this review, we summarized studies related to cell migration and regulation of this process by Cxs. The information exposed here strongly suggests that astrocyte reactivity, as well as migration in a pro-inflammatory environment, relies predominantly on Cx HCs, rather than GJs.

Despite the similarity between Cxs, all of them show different properties that provide a broad spectrum of responses in any given situation. However, Cx43 probably emerges as the main Cx involved in astrocyte physiology, controlling its reactive phenotype, allowing migration and facilitating cell-cell communication with surrounding cells.

The effect of Cxs on migration is usually observed during inflammation, and the presence of Cx HCs is required to maintain the reactive phenotype of astrocytes after injury. Inhibition of Cx HCs with peptides or blockade of the P2X7R improves function after spinal cord injury or EAE symptoms, respectively, indicating that the modulation of this signaling pathway could provide a therapeutic opportunity to treat these conditions. Release of ATP by Cx HCs and intake of Ca^2+^ through the P2X7R are among the crucial steps for astrocyte reactivity and migration, demonstrating that these two processes are closely related, since only reactive astrocytes migrate.

The participation of Cxs in astrocyte migration is related to their function as channels and the communication that they mediate through cell-cell, as well as cell-ECM interactions. Importantly, the regulation of cell adhesion and cytoskeletal dynamics, both of which are relevant to cell migration, occurs by post-translational modifications of Cxs, which are induced by kinases, phosphatases, and acetylases. In this scenario, both phosphorylation and acetylation regulate membrane localization of Cxs.

It is easy to speculate that GJs are not related to the maintenance of the negative conditions that characterize astrogliosis, since they exert their role in cell-cell communication and not in cell-ECM communication. We believe that single-cell migration is the operating mechanism during astrogliosis, and considering that GJs are reportedly more important for collective migration, and that this type of cell movement is not observed in the CNS after injury (Carbonell, 2005; Retamal, 2007), we propose that mostly HCs, rather than GJs, are related to astrocyte migration.

The presence of Cx HCs in astrocytes is not only important for the initiation of reactivity or migration, but also to maintain the reactive phenotype during longer periods, which then increases the negative effects of neurological diseases or pro-inflammatory conditions. This Cx role has been supported by several groups and could represent an important target for treatment or prevention of such pathologies. However, due to the importance of Cx HCs in astrocyte reactivity and migration, a specific treatment based on HC blockade should be pursued, especially for neurodegenerative diseases and astrocytoma/glioma treatment.

New studies able to discriminate between the two distinct Cx channel activities are necessary to enlighten the specific Cx roles in physiological and pathological conditions and for future development of interventions that will be able to ameliorate the detrimental effects of CNS injury and neurodegenerative diseases. The challenge will be to modulate reactive astrocytes according to the optimal regenerative responses desired, and to define the correct therapeutic window according to the specific stage of the pathology. These future therapeutic strategies should consider both pharmacological and nonpharmacological approaches to enrich the environment necessary for CNS regeneration (Pekny and Pekna, 2014). Furthermore, future research on the complex molecular mechanisms that regulate astrocyte migration is also needed for the development of clinical interventions for astrogliosis.

## Author Contributions

LL contributed in the conception and design of the work; FB-B and RL-C to the acquisition, analysis, and interpretation of data for the work. AA, RL-C, and LL contributed drafting the work. All authors critically revised the work for important intellectual content, provided approval for publication of the content, and agreed to be accountable for all aspects of the work.

## Funding

LL was supported by Fondo Nacional de Desarrollo Científico y Tecnológico #1150744 (LL) and Comisión Nacional de Investigación Científica y Tecnológica-FONDAP #15130011 (LL). Fondo Nacional de Desarrollo Científico y Tecnológico #3140460 (RL-C). AA would like to thank the support of Project DIUA 169-2019 from Universidad Autónoma de Chile.

## Conflict of Interest

The authors declare that the research was conducted in the absence of any commercial or financial relationships that could be construed as a potential conflict of interest.
